# Can Left Atrioventricular Valve Reduction Index (LAVRI) Predict the Surgical Strategy for Repair of Atrioventricular Septal Defect?

**DOI:** 10.1007/s00246-021-02558-5

**Published:** 2021-02-12

**Authors:** Anastasia Schleiger, Peter Kramer, Marie Schafstedde, Mustafa Yigitbasi, Friederike Danne, Peter Murin, Mi-Young Cho, Joachim Photiadis, Felix Berger, Stanislav Ovroutski

**Affiliations:** 1 Department of Congenital Heart Disease/Pediatric Cardiology, German Heart Center Berlin, Augustenburger Platz 1, 13353 Berlin, Germany; 2 Berlin Institute of Health, Charité Universitätsmedizin Berlin, Berlin, Germany; 3 Department of Congenital Heart Surgery/Pediatric Heart Surgery, German Heart Center Berlin, Berlin, Germany; 4 Department of Pediatrics, Division of Cardiology, Charité Universitätsmedizin Berlin, Berlin, Germany

**Keywords:** Atrioventricular septal defect, Echocardiographic analysis, Preoperative decision-making, Left atrioventricular valve repair, Unbalanced atrioventricular septal defect

## Abstract

Despite improved survival, surgical treatment of atrioventricular septal defect (AVSD) remains challenging. The optimal technique for primary left atrioventricular valve (LAVV) repair and prediction of suitability for biventricular approach in unbalanced AVSD are still controversial. We evaluated the ability of our recently developed echocardiographic left atrioventricular valve reduction index (LAVRI) in predicting LAVV reoperation rate and surgical strategy for unbalanced AVSD. Retrospective echocardiographic analysis was available in 352 of 790 patients with AVSD treated in our institution and included modified atrioventricular valve index (mAVVI), ventricular cavity ratio (VCR), and right ventricle/left ventricle (RV/LV) inflow angle. LAVRI estimates LAVV area after complete cleft closure and was analyzed with regard to surgical strategy in primary LAVV repair and unbalanced AVSD. Of the entire cohort, 284/352 (80.68%) patients underwent biventricular repair and 68/352 (19.31%) patients underwent univentricular palliation. LAVV reoperation was performed in 25/284 (8.80%) patients after surgical correction of AVSD. LAVRI was significantly lower in patients requiring LAVV reoperation (1.92 cm^2^/m^2^ [IQR 1.31] vs. 2.89 cm^2^/m^2^ [IQR 1.37], *p* = 0.002) and significantly differed between patients receiving complete and no/partial cleft closure (2.89 cm^2^/m^2^ [IQR 1.35] vs. 2.07 cm^2^/m^2^ [IQR 1.69]; *p* = 0.002). Of 82 patients diagnosed with unbalanced AVSD, 14 were suitable for biventricular repair (17.07%). mAVVI, LAVRI, VCR, and RV/LV inflow angle accurately distinguished between balanced and unbalanced AVSD and predicted surgical strategy (all *p* < 0.001). LAVRI may predict surgical strategy in primary LAVV repair, LAVV reoperation risk, and suitability for biventricular approach in unbalanced AVSD anatomy.

**Supplementary Information:**

The online version contains supplementary material available at 10.1007/s00246-021-02558-5.

## Introduction

Due to advances in surgical techniques, postoperative management, and pre- and intraoperative echocardiographic imaging, survival rates after surgical correction of atrioventricular septal defect (AVSD) significantly increased over the past decades [1–3]. Nevertheless, regurgitation of the left atrioventricular valve (LAVV) represents the major cause for reoperation, long-term morbidity, and mortality [3–5]. Associated cardiovascular anomalies, malformations of the LAVV, infeasible primary complete cleft closure and normal karyotype have been described as risk factors for reoperation [3–6]. Additionally, the degree of LAVV regurgitation after AVSD repair strongly correlates with the indication for subsequent LAVV reconstruction or replacement [3, 4]. Therefore, the preoperative evaluation of atrioventricular valve anatomy seems indispensable for devising the optimal surgical strategy of primary LAVV repair and predicting the risk for LAVV reoperation.

Another challenge concerning AVSD repair is the surgical management of unbalanced AVSD due to uncertainties in decision-making between biventricular repair (BVR) and univentricular palliation (UVP) in borderline AVSD anatomy. Unbalanced AVSD is commonly defined by ventricular hypoplasia, malalignment of the atrioventricular junction and atrioventricular valve dysplasia [7]. Compared to patients with balanced AVSD, in patients with unbalanced anatomy, BVR is associated with a higher mortality rate and a more complicated postoperative course including numerous re-interventions [7–11]. Although various echocardiographic indices have been introduced to facilitate decision-making, so far no universal recommendations exist to unequivocally select the optimal therapeutic approach in case of ventricular imbalance.

In this study we introduce our recently developed left atrioventricular valve reduction index (LAVRI) for preoperative echocardiographic assessment in patients with AVSD. We evaluated the ability of LAVRI in devising surgical strategy in primary LAVV repair and predicting the LAVV reoperation risk. Additionally, we performed a detailed retrospective echocardiographic analysis measuring ventricular septal defect (VSD) size, modified atrioventricular valve index (mAVVI), ventricular cavity ratio (VCR), right ventricular/ left ventricular (RV/LV) inflow angle, and LAVRI to analyze and compare the usability of LAVRI in predicting and defining surgical strategy in unbalanced AVSD anatomy.

## Patients and Methods

### Study Design and Patient Cohort

We retrospectively identified 790 patients diagnosed with AVSD in our institution. All patients who underwent surgical correction of AVSD or palliation before January 2006 were excluded due to the unavailability of digitally archived echocardiograms for retrospective analysis (*n* = 419). Fourteen patients, who did not receive corrective surgery in our institution, but were referred from other centers for reoperation, were excluded from further analysis. Additional five patients were excluded, since surgical correction was not performed until conclusion of data acquisition. Echocardiograms and medical records of 352 patients were reviewed for this study. Median follow-up after AVSD repair was 6.21 years [IQR 7.34]. Unbalanced AVSD was defined as the presence of hypoplastic or non-apex forming ventricles and/ or an atrioventricular valve override of > 60% over either ventricle [11]. Parameters extracted from postoperative course included total hospital stay, ICU stay, and ventilation time. The study was approved by the institutional review board and ethics committee (decision number: EA2/127/16). Informed consent was not considered mandatory due to the retrospective character of this study.

### Echocardiograpic Evaluation

**Fig. 1:**
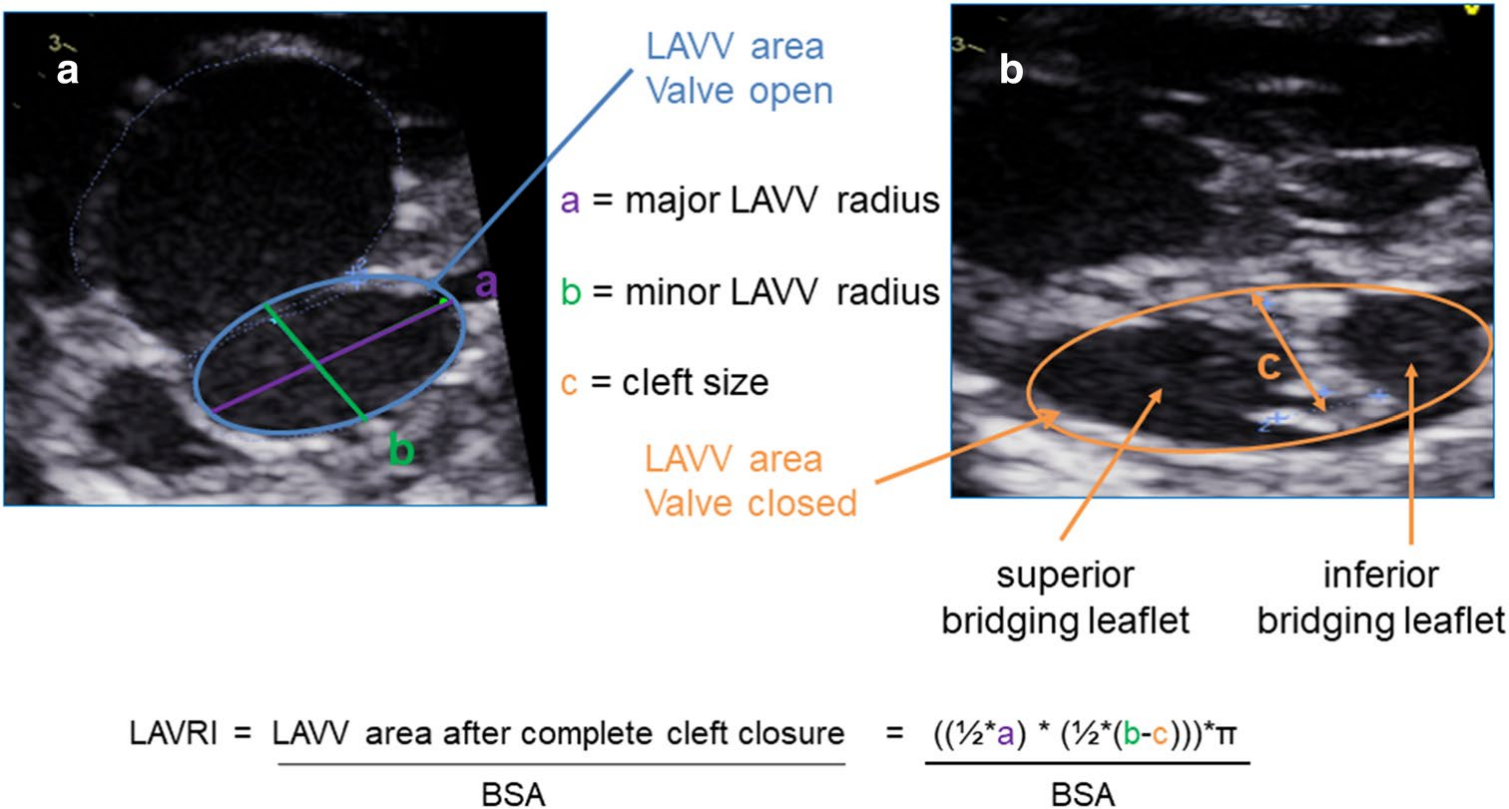
Measurement and calculation of LAVRI. Left anterior oblique view of the common AV valve at end-diastole (**a**) and systole (**b**) in a patient with right-dominant unbalanced AVSD. Measurements required for LAVRI calculation: a = major LAVV radius, b = minor LAVV radius, and c = cleft size. *LAVV* left atrioventricular valve area, *LAVRI* left atrioventricular valve reduction index

**Fig. 2:**
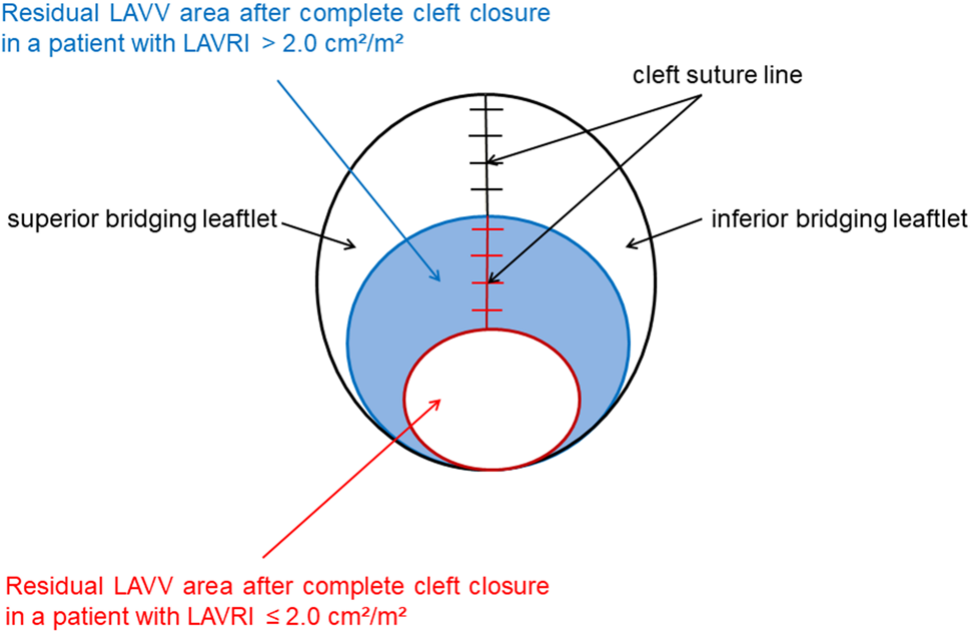
Schematic drawing of the residual LAVV orifice after complete cleft closure in patients with a LAVRI ≤ 2.0 cm^2^/m^2^ (red) and > 2.0 cm^2^/m^2^ (blue). *LAVV* left atrioventricular valve area, *LAVRI* left atrioventricular valve reduction index

Echocardiographic analysis was performed retrospectively on digitally archived routine pre- and postoperative echocardiograms. Parameters assessed included mAVVI, VCR, RV/LV inflow angle, and our developed LAVRI. mAVVI was obtained by visualizing the orifice of the common AV valve in the left anterior oblique view at end-diastole and bisecting the common AV valve along a connecting line from muscular to infundibular ventricular septum [5]. The mAVVI was calculated by dividing the left AVV area by the total AVV area [5]. VCR was calculated as the ratio between the left ventricular length multiplied with the left ventricular width and the right ventricular length multiplied with the right ventricular width [8]. Ventricular length and width were measured in apical four-chamber view from the AV valve to the apex and from ventricular septum to the lateral wall of each ventricle [8]. RV/LV angle was measured in the apical four-chamber view from the crest of the ventricular septum as the apex to each atrioventricular valve hingepoint [12]. LAVRI was developed by our study group to predict resulting LAVV area after complete cleft closure using a modified formula for the calculation of an ellipse area: A = πab; A = area, a = major LAVV radius, b = minor LAVV radius minus cleft size. Major and minor LAVV radius were measured in the left anterior oblique view of the common AV valve at end-diastole after bisecting the common AV valve along the line from muscular to infundibular ventricular septum into left and right AVV area (Fig. 1). Cleft size was measured from the coaptation zone of the left mural, superior, and inferior bringing leaflet to a line corresponding to the plane of the interventricular septum in the left anterior oblique view (Fig. 1). LAVRI was obtained by indexing predicted resulting LAVV area after complete cleft closure to body surface area calculated from the Du Bois formula (Fig. 2). Postoperative AV valve regurgitation was classified as absent/ mild, moderate and severe by visual assessment of the regurgitation jet dimensions in color Doppler sonography. Additionally, mean inflow pressure gradient was determined from Doppler velocity flow curve of the left and right AV valve. Echocardiograms were evaluated by one echocardiographer (AS) and measurements were verified by a second echocardiographer (SO). Both echocardiographers were blinded to the surgical approach. All measurements were obtained in three cardiac cycles and values averaged for final analysis. Echocardiography was performed using two echocardiographic devices (Vivid 7 and E 9, GE Healthcare, Solingen, Germany). Xcelera V (Philips Healthcare, Eindhoven, The Netherlands) was used for retrospective echocardiographic measurements.

### Surgical Technique of AVSD Repair

Our institutional technique of AVSD repair has previously been described in detail [3]. Briefly, all patients were placed on cardiopulmonary bypass with aortic and bicaval cannulation in moderate hypothermia (32 °C rectal temperature). Cardioplegic arrest was achieved using Kirsch/ hydroxyethyl starch or Bretschneider cardioplegic solution. Based on AVSD anatomy, corrective surgery was performed using two-patch technique in 177 patients, single-patch technique in 18 patients, patch closure of the atrial septum and direct closure of the ventricular septum (or vice versa) in 54 patients and sole patch closure of the atrial septum in 35 patients. Two-hundred forty-seven patients underwent complete and 37 patients no/ partial cleft closure.

### Statistical Analysis

Data were obtained from medical records of the German Heart Centre Berlin. Patients characteristics are expressed as median and interquartile range [IQR] calculated as 75th minus 25th percentile. Patients` characteristics and echocardiographic indices were compared using chi-square test for categorical variables and Mann–Whitney *U* test for continuous variables. Predictability for surgical strategy of echocardiographic indices was analyzed using receiver operating characteristic (ROC) curves. Statistical analysis was performed using SPSS statistical software program (version 23, IBM Corp., NY, USA). A *p-*value < 0.05 was considered statistically significant.

## Results

### Patient Characteristics

Patient characteristics of the entire cohort are listed in Table 1. Of 352 consecutive patients operated in the past 14 years, 284 underwent surgical repair of AVSD and 68 received univentricular palliation, respectively. In the BVR subgroup 244 patients were diagnosed with complete, 35 patients with intermediate, and 5 patients with partial AVSD. Trisomy 21 was present in 168 patients. Median patient age at corrective surgery was 5.20 months [IQR 3.91] and median patient weight 5.44 kg [IQR 2.40]. Forty-eight patients presented with complex AVSD including the following associated cardiac malformations: right ventricular outflow tract obstruction/ pulmonary atresia (*n* = 16), coarctation of the aorta/interrupted aortic arch (*n* = 23), total or partial anomalous pulmonary venous connection (*n* = 9), double outlet right ventricle (*n* = 4), heterotaxy (*n* = 6) or malposition of the great arteries (*n* = 2).

**Table 1:** Patient characteristics

Characteristic, *n* (%)/[IQR]	Entire cohort	BVR	UVP	*p-*value
Number of patients	352	284	68	
Male	147 (41.76)	116 (40.84)	31 (45.59)	0.496
Trisomy 21	168 (47.73)	168 (59.15)	0 (0.0)	** < 0.001**
Patient weight (kg)^a^	5.40 [2.68]	5.44 [2.40]	5.23 [5.15]	0.177
BSA (m^2^)	0.30 [0.09]	0.30 [0.07]	0.29 [0.19]	0.245
Patient age (months)^a^	5.64 [07.69]	5.16 [3.96]	31.7 [38.07]	** < 0.001**
AVSD type				
Complete	312 (88.64)	244 (85.92)	68 (100.0)	** < 0.001**
Intermediate	35 (9.94)	35 (12.32)	0 (0.0)	**0.001**
Partial	5 (1.42)	5 (1.76)	0 (0.0)	0.588
Complex AVSD	110 (31.25)	48 (16.90)	62 (91.18)	** < 0.001**
RVOTO/PA	51 (14.49)	16 (5.63)	35 (51.47)	** < 0.001**
CoA/AAH	37 (10.51)	23 (8.10)	14 (20.59)	**0.007**
TAPVD/PAPVD	37 (10.51)	9 (3.17)	28 (41.18)	** < 0.001**
DORV	23 (6.53)	4 (1.41)	19 (27.94)	** < 0.001**
Heterotaxy	39 (11.08)	6 (2.11)	33 (48.53)	** < 0.001**
TGA	39 (11.08)	2 (0.70)	37 (54.41)	** < 0.001**

Statistically significant results are given in bold letters

Data are presented as median [IQR] or frequencies (%)

*AAH* hypoplastic aortic arch, *AVSD* atrioventricular septal defect, *BSA* body surface area, *BVR* biventricular repair, *CoA* aortic coarctation, *DORV* double outlet right ventricle, *PAPVD* partial abnormal pulmonary venous drainage, *RVOTO/PA* right ventricular outflow tract obstruction/pulmonary atresia, *TAPVD* total abnormal pulmonary venous drainage, *TGA* transposition of the great arteries, *UVP* univentricular palliation

^a^Patient age and weight refer to the date of echocardiographic examination, which was used for retrospective analysis and measurement of indices

### Surgical Strategy in Balanced AVSD

Of 284 patients who received BVR, primary LAVV repair was performed with complete cleft closure in 247 patients and partial cleft closure in 28 patients. In 9 patients, cleft closure was not feasible due to abnormal LAVV anatomy or unfavorably small LAVV size to avoid postoperative stenosis.

**Fig. 3:**
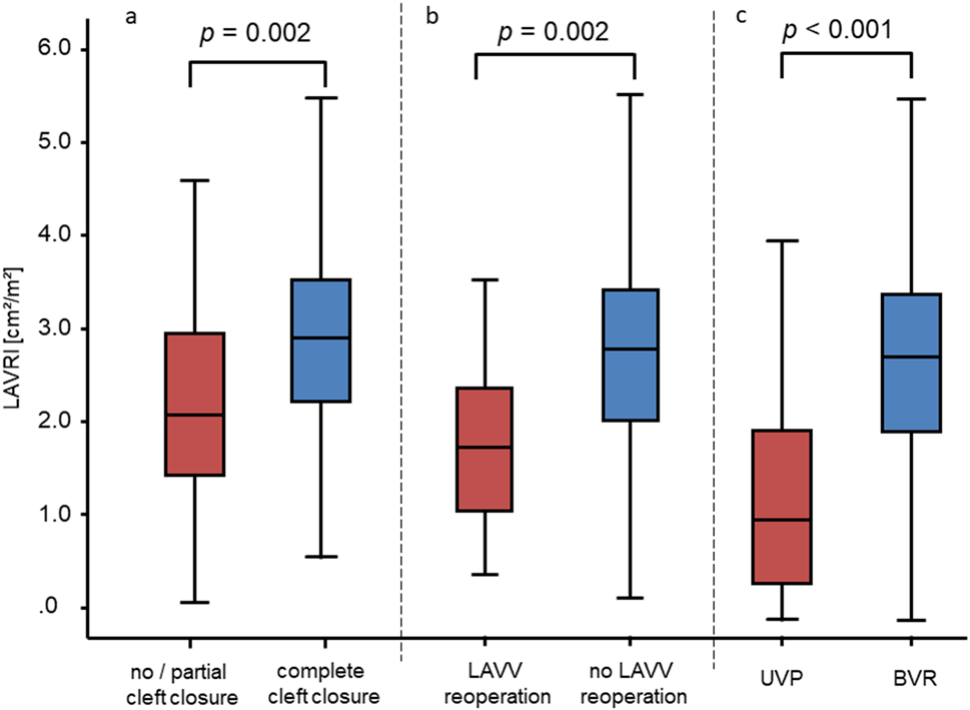
**a** LAVRI according to strategy for primary LAVV repair. Patients are divided into two groups based on surgical strategy: Group 1: No/ partial cleft closure (*n* = 29), group 2: complete cleft closure (*n* = 142). **b** LAVRI according to requirement of LAVV reoperation. Patients are divided into patients with (*n* = 16) and patients without reoperation (*n* = 155). **c** LAVRI according to surgical strategy. Patients are divided into two groups: Group 1 UVP (*n* = 46), Group 2 BVR (*n* = 171). *LAVRI* left atrioventricular valve reduction index

Preoperative LAVRI measurement was retrospectively available in 171 of 284 patients (60.21%). LAVRI was significantly lower in patients receiving primary LAVV repair with no/partial cleft closure compared to patients receiving complete cleft closure (2.07 cm^2^/m^2^ [IQR 1.69] vs. 2.89 cm^2^/m^2^ [IQR 1.35]; *p* = 0.002, Fig. 3a). Patients with no/ partial cleft closure required LAVV reoperation significantly more frequent than patients with complete cleft closure (*n*_1_ = 8/36 (22.22%) vs. *n*_2_ = 17/248 (6.85%); *p* = 0.007). Twenty-five patients required a reoperation addressing the LAVV. Of these, 21 patients underwent secondary LAVV repair and 4 patients LAVV replacement. After initial re-repair of the LAVV, 4 patients required subsequent valve replacement after a median follow-up of 10.22 months [IQR 2.01]. According to intraoperative findings, major causes of moderate or severe LAVV regurgitation were dehiscence of the cleft suture (*n* = 8), partial cleft closure (*n* = 12), infeasible primary repair of double orifice (*n* = 6) or leaflet prolapse (*n* = 1). Most common reoperation techniques were redo cleft closure (*n* = 17), annuloplasty (*n* = 3) or commissuroplasty (*n* = 3). AVSD type (complete, intermediate, partial) was not associated with requirement of LAVV reoperation *(p* = 0.781). LAVV regurgitation at discharge after AVSD repair or before in-hospital reoperation was graded absent/ mild in 205 patients, moderate in 70 patients, and severe in 9 patients. Expectedly, the degree of LAVV regurgitation strongly correlated with the necessity of LAVV reoperation (*p* < 0.001). LAVRI significantly differed between patients with absent/ mild and patients with moderate/ severe LAVV regurgitation at discharge (3.0 cm^2^/m^2^ [IQR 1.51] vs. 2.53 cm^2^/m^2^ [IQR 1.34], *p* = 0.007). Median LAVV inflow pressure gradient at discharge did not significantly differ between patients with and without requirement for LAVV reoperation (1.80 mmHg [IQR 2.16] vs. 1.67 mmHg [IQR 1.53], *p* = 0.20).

LAVRI was significantly lower in patients requiring LAVV reoperation compared to patients without an indication for reoperation (1.92 cm^2^/m^2^ [IQR 1.31] vs. 2.91 cm^2^/m^2^ [IQR 1.37], *p* = 0.002, Fig. 3b). Patients with an LAVRI ≤ 2.03, equalling the 25th percentile of the BVR cohort, had a significantly higher risk for a reoperation addressing the LAVV (Odds Ratio: 5.79 [95% CI 1.99–16.86], *p* = 0.002).

### Surgical Strategy in Unbalanced AVSD

**Fig. 4:**
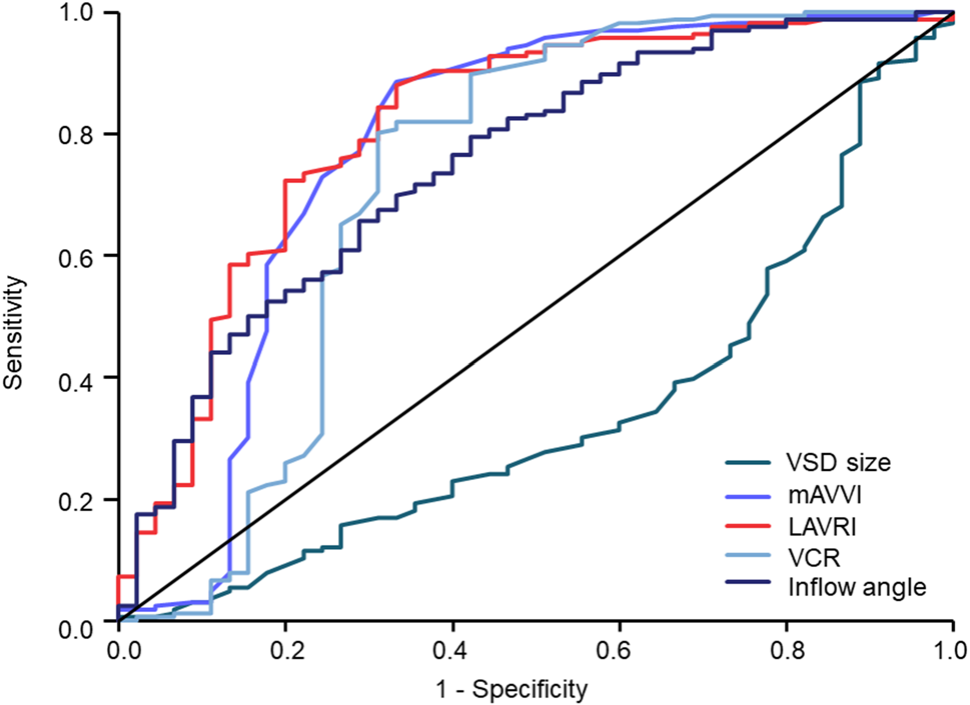
Receiver operating curve analysis of each echocardiographic index concerning prediction of surgical strategy. *mAVVI *modified atrioventricular valve index, *BVR* biventricular repair, *LAVRI* left atrioventricular valve reduction index, *RV/LV* right ventricular/left ventricular, *VCR* ventricular cavity ratio, *VSD* ventricular septal defect

Of 68 patients undergoing UVP, 34 patients were palliated with Fontan completion and 34 patients were interstage including 21 patients with superior cavopulmonary anastomosis and 13 patients with an aortopulmonary shunt at last follow-up. Forty-six patients were characterized by right ventricular dominance and twelve patients by left ventricular dominance. In the UVP cohort, complex AVSD was diagnosed significantly more frequent than in the BVR cohort: Right ventricular outflow tract obstruction/ pulmonary atresia occurred in 35 patients, coarctation of the aorta/interrupted aortic arch in 14 patients, total or partial anomalous pulmonary venous connection in 28 patients, double outlet right ventricle in 19 patients, heterotaxy in 33 patients, and malposition of the great arteries in 37 patients (all *p* ≤ 0.007, Table 1). Echocardiographic parameters and indices comparing patients who underwent BVR and UVP are listed in Table 2: LAVRI measurement was available in 46 of 68 patients (67.65%) and significantly differed between patients receiving UVP and BVR (1.18 cm^2^/m^2^ [IQR 1.64] vs. 2.80 cm^2^/m^2^ [IQR 1.44], *p* < 0.001, Table 2, Fig. 3c). In patients with right-dominant unbalanced AVSD median LAVRI was 0.83 cm^2^/m^2^ [IQR 1.12] and 3.24 cm^2^/m^2^ [IQR 1.59] in patients characterized by left ventricular dominance (p < 0.001). VSD size, mAVVI, VCR, and RV/LV inflow angle significantly diverged between patients undergoing BVR or UVP (Table 2). ROC analysis showed acceptable discrimination between surgical strategies for LAVRI, mAVVI, VCR, and inflow angle and unsatisfying discrimination for VSD size (Table 3, Fig. 4). Based on area under the curve (AUC) calculations, the accuracy of LAVRI in discriminating between BVR and UVP was superior to the other echocardiographic indices (Table 3, Fig. 4).

**Table 2:** Echocardiographic parameters according to surgical strategy

Echocardiographic parameter/index	BVR	UVP	*p*-value
VSD size (cm)	0.73 [0.53]	0.89 [0.41]	** < 0.001**
mAVVI	0.49 [0.08]	0.35 [0.17]	** < 0.001**
LAVRI (cm^2^/m^2^)	2.80 [1.44]	1.18 [1.64]	** < 0.001**
VCR	0.93 [0.36]	0.55 [0.52]	** < 0.001**
RV/LV inflow angle (°)	97.71 [28.99]	75.71 [26.07]	** < 0.001**

Statistically significant results are given in bold letters

Data are presented as median [IQR]

*mAVVI* modified atrioventricular valve index, *BVR* biventricular repair, *LAVRI* left atrioventricular valve reduction index, *RV/LV* right ventricular/left ventricular, *UVP* univentricular palliation, *VCR* ventricular cavity ratio, *VSD* ventricular septal defect

**Table 3:** Area under the curve from receiver operating characteristic curve analysis of echocardiographic indices with regard to discrimination between BVR and UVP

Parameter	AUC	*p*-value	95% CI
VSD size (cm)	0.329	** < 0.001**	0.238–0.420
mAVVI	0.782	** < 0.001**	0.685–0.878
LAVRI (cm^2^/m^2^)	0.792	** < 0.001**	0.709–0.878
VCR	0.721	** < 0.001**	0.614–0.828
RV/LV inflow angle (°)	0.756	** < 0.001**	0.675–0.836

Statistically significant results are given in bold letters

*AUC* area under the curve, *BVR* biventricular repair, *CI* confidence interval, *LAVRI* left atrioventricular valve reduction index, *mAVVI* modified atrioventricular valve index, *RV/LV* right ventricular/left ventricular, *UVP* univentricular palliation, *VCR* ventricular cavity ratio, *VSD* ventricular septal defect

Fourteen patients diagnosed with unbalanced AVSD were considered suitable for biventricular approach: Twelve patients received primary biventricular repair and 2 patients biventricular conversion from prior single ventricle palliation (one-and-a-half biventricular repair with surgical correction of AVSD and leaving the superior cavopulmonary anastomosis in one and staged conversion with initial repair of total anomalous pulmonary venous return, secondary Kawashima operation and final Kawashima take-down and surgical AVSD repair in the other patient). Ten patients were characterized by right ventricular dominance and four patients by left ventricular dominance. Mortality and LAVV reoperation rate did not significantly differ between patients with balanced and unbalanced AVSD receiving BVR (*p*_1_ = 0.360; *p*_2_ = 0.358). In patients with unbalanced AVSD, partial or no cleft closure was performed significantly more frequent than in patients with balanced AVSD (*n*_1_ = 7/14 (50.0%) vs. *n*_2_ = 29/270 (10.74%), *p* = 0.001). In this particular patient cohort, LAVRI was significantly lower than in patients with balanced AVSD anatomy receiving BVR (1.55 cm^2^/m^2^ [IQR 2.15] vs. 2.87 cm^2^/m^2^ [IQR 1.37], *p* = 0.002). Postoperative course did not significantly vary between patients with balanced and unbalanced AVSD receiving BVR in terms of total hospital and intensive care unit (ICU) stay (total hospital stay: 10 days [IQR 8.0] vs. 12.0 days [IQR 9.0], *p*_1_ = 0.390, ICU stay: 5.0 days [IQR 6.0] vs. 6.0 days [IQR 7.0], *p*_2_ = 0.092). In patients with unbalanced AVSD a trend toward longer ventilation time was observed (105.0 h [IQR 183.0] vs. 53.0 h [IQR 111.75], *p* = 0.042].

## Discussion

Despite improved survival rates of patients with AVSD, two major issues remain controversial concerning surgical AVSD repair: First, the reoperation rate of the LAVV and second the suitability for biventricular approach in unbalanced AVSD anatomy. In this study we introduced the left atrioventricular valve reduction index (LAVRI) for preoperative estimation of the resulting left atrioventricular valve area after complete cleft closure. We evaluated the usability of LAVRI in facilitating surgical decision-making concerning primary LAVV repair, estimating the risk of LAVV reoperation and predicting the suitability for biventricular approach in unbalanced AVSD.

### Primary LAVV Repair and Risk of LAVV Reoperation

Late outcomes after surgical correction of AVSD are compromised by the substantial risk for reoperation of the LAVV, which results in considerable mortality and morbidity of this particular patient cohort [1–4]. Normal karyotype, moderate or severe pre– and postoperative LAVV regurgitation, LAVV dysplasia, and double orifice have been identified as risk factors for LAVV reoperation [1–4, 13]. Despite primary LAVV repair with complete cleft closure, deterioration of the LAVV competence is common after AVSD repair and contributes to an increased risk for reoperation [14]. In our cohort, LAVV reoperation occurred in 8.80% of patients after AVSD repair. Major causes for moderate or severe LAVV regurgitation requiring reoperation were dehiscence of the cleft suture, incomplete cleft closure or LAVV anomalies (such as double orifice and deficient mural or bridging leaflets), which were not addressed during primary LAVV repair. Complete cleft closure is generally favored to minimize reoperation risk, but pre- and intraoperative decision-making remains challenging since the LAVV apparatus is still fragile and prone to laceration at the age of 3 to 6 months, when AVSD repair is usually performed [1–4, 14]. Depending on atrioventricular valve morphology, equilibrating the optimal balance between residual LAVV regurgitation and a stenotic valve function may represent an enormous technical challenge. Our retrospective analysis revealed the feasibility of our developed LAVRI in assessing surgical options of primary LAVV repair: In patients with a LAVRI > 2.0 cm^2^/m^2^ the cleft was preferably closed completely, whereas in patients with a LAVRI ≤ 2.0 cm^2^/m^2^, LAVV cleft could only be closed partially or not closed at all (Fig. 2). In this patient cohort LAVV reoperation occurred significantly more frequent due to the development of moderate or severe LAVV regurgitation during follow-up. Additionally, a LAVRI ≤ 2.0 cm^2^/m^2^ was associated with an almost sixfold higher risk for LAVV reoperation. The preoperative prediction of an increased reoperation risk in patients with a LAVRI ≤ 2.0 cm^2^/m^2^ might be useful to provide more detailed information concerning the expected long-term outcome and prepare parents and treating physicians for a possible need of a LAVV reoperation after initial repair.

### Suitability for BVR in Unbalanced AVSD

Due to the wide spectrum of unbalanced AVSD anatomy, no universal consensus exists, which parameters define the preferable surgical strategy and facilitate decision-making concerning the feasibility of biventricular approach [7, 15, 16]. Since mortality rates of patients with unbalanced AVSD undergoing single ventricle palliation are substantial, a biventricular repair should be favored [17, 18]. Del Nido et al. demonstrated a significant survival benefit in patients who received biventricular conversion from prior single ventricle palliation or staged biventricular recruitment compared to patients with definite univentricular palliation, even at the expense of a significantly higher number of post-surgical re-operations and re-interventions [11].

Since feasibility of biventricular repair depends on various anatomic features, such as ventricular size, AV valve morphology, inflow geometry, VSD size or associated cardiac malformations, a single echocardiographic index alone cannot adequately determine the appropriateness of surgical strategy. Generally, all indices evaluated in our study, except VSD size, proved satisfying identifiers of the magnitude of ventricular imbalance and were strongly associated with surgical strategy. Two fundamental factors seem crucial for BVR in unbalanced AVSD: First, AVSD anatomy including the size of left and right ventricular cavity and the extent of the disproportion of the atrioventricular junction and second the associated cardiac malformations.

The introduced LAVRI proved usable in predicting surgical strategy for primary LAVV repair in balanced AVSD and suitability for biventricular approach in unbalanced AVSD. In patients with unbalanced AVSD with dominant right ventricle receiving BVR, complete cleft closure was not feasible due to a small LAVV area (LAVRI ≤ 2.0 cm^2^/m^2^). These patients received primary LAVV repair with no or only partial cleft closure since the cleft was frequently the only component of the LAVV opening area. In these patients, frequency of LAVV reoperation was not higher compared to patients with balanced AVSD repaired with only partial or without cleft closure (*n*_1_ = 1/7 (14.29%) vs. *n*_2_ 7/29 (24.14%), *p* = 0.503).

## Conclusion

The introduced LAVRI is strongly associated with surgical strategy for balanced and unbalanced AVSD and may, therefore, prove valuable in the planning of surgical procedures and the prediction of postoperative results. Moreover, it adequately differentiates between ventricular imbalance suitable or unsuitable for biventricular repair. Surgical decision-making for biventricular approach in unbalanced AVSD cannot be based on one single index but combining LAVRI with previously reported echocardiographic indices might substantially increase accuracy of preoperative assessment and in turn result in improved surgical outcomes.

## Limitations

There are several limitations to this study. This is a retrospective, single-center study with a small patient cohort. Further studies, preferably with a larger patient cohort and in a multi-center setting, are needed to evaluate the suitability of LAVRI in defining surgical strategy. The number of patients with unbalanced AVSD receiving BVR in our study is small; results of morbidity and mortality after BVR might change in a larger patient cohort. The skewed distribution of patients receiving UVP and BVR might confound the capacity of echocardiographic indices in defining surgical strategy. Additionally, no ventricular growth strategies were performed in our institution. Therefore, decision-making for BVR in unbalanced AVSD was performed based on preoperatively preexistent anatomic features without consideration of possible growth potential. Additionally, MRI analysis and measurement of ventricular end-diastolic volumes was not performed in this study, which is only based on echocardiographic evaluation of ventricular size and AV valve disproportion.

## Supplementary Information

Below is the link to the electronic supplementary material.

Supplementary Information 1 (XLSX 24 kb)

## Abbreviations

AVSD: Atrioventricular septal defect
(L)AVV: (Left) atrioventricular valve
BVR: Biventricular repair
UVP: Univentricular palliation
LAVRI: Left atrioventricular valve reduction index
VSD: Ventricular septal defect
mAVVI: Modified atrioventricular valve index
VCR: Ventricular cavity ratio
RV/LV: Right ventricle/ left ventricle
IQR: Interquartile range
ROC: Receiver operating characteristic
Vs.: Versus
AUC: Area under the curve
CI: Confidence interval
ICU: Intensive care unit
h: Hours
